# Effect of Drought Stress and Osmopriming on the Growth and Yield of Tadong Upland Rice in Sabah, Malaysia

**DOI:** 10.21315/tlsr2026.37.1.10

**Published:** 2026-03-31

**Authors:** Robin Ah Hee Lim, Evelyn Shin Rou Koay, Mok Sam Lum

**Affiliations:** 1 Faculty of Tropical Forestry, Universiti Malaysia Sabah, Jalan UMS, 88400 Kota Kinabalu, Sabah, Malaysia; 2 Faculty of Sustainable Agriculture, Universiti Malaysia Sabah, Locked Bag No. 3, P. O. Box No. 3, 90509 Sandakan, Sabah, Malaysia

**Keywords:** Tadong Upland Rice, Polyethylene Glycol, Osmopriming, Drought, Rice Yield, Osmotic Potential, Padi Bukit Tadong, Polietilena Glikol, Osmopriming, Kemarau, Hasil Padi, Potensi Osmotik

## Abstract

Drought stress remains one of the most significant challenges facing the development and production of upland rice. Seed osmopriming represents a method to enhance plant growth and offers potential against drought stress. This study aimed to investigate the effects of polyethylene glycol (PEG) 6000 seed osmopriming and drought stress on the growth, yield, physiological and biochemical parameters of Tadong upland rice. The growth and yield of the Tadong upland rice variety were evaluated at five osmotic potentials using PEG 6000 for drought-induced osmopriming and at varying levels of drought stress with three soil moisture contents (SMC). Significant interaction effects between PEG 6000 osmotic potential levels and drought stress levels were observed across all measured parameters of Tadong upland rice. The combination of 20% SMC with –4 bar PEG proved optimal for growth and yield, showing a 55.68% increase compared to 10% SMC with −2 bar PEG, which produced the lowest yield per plant. Additional research should examine the effects of different PEG 6000 osmotic potential and drought stress levels on other upland rice varieties.

## Abstrak

Tekanan kemarau kekal sebagai salah satu cabaran paling ketara yang dihadapi oleh pembangunan dan pengeluaran padi bukit. Osmopriming benih mewakili kaedah untuk meningkatkan pertumbuhan tumbuhan dan menawarkan potensi mengatasi tekanan kemarau. Kajian ini bertujuan untuk mengkaji kesan osmopriming biji benih polietilena glikol (PEG) 6000 dan tekanan kemarau terhadap parameter pertumbuhan, hasil, fisiologi dan biokimia padi bukit Tadong. Pertumbuhan dan hasil varieti padi bukit Tadong diuji pada lima potensi osmotik menggunakan PEG 6000 untuk osmopriming akibat kemarau dan pada tahap tekanan kemarau yang berbeza-beza dengan tiga kandungan lembapan tanah (SMC). Kesan interaksi yang ketara antara tahap potensi osmotik PEG 6000 dan tahap tekanan kemarau diperhatikan merentas semua parameter yang diukur padi tanah tinggi Tadong. Gabungan 20% SMC dengan −4 bar PEG terbukti optimum untuk pertumbuhan dan hasil menunjukkan peningkatan 55.68% berbanding 10% SMC dengan −2 bar PEG, yang menghasilkan hasil terendah bagi setiap tumbuhan. Penyelidikan tambahan harus mengkaji kesan potensi osmotik PEG 6000 yang berbeza dan tahap tekanan kemarau pada varieti padi bukit yang lain.

**HIGHLIGHTS**

- Growth, yield, physiological and biochemical parameters of Tadong upland rice were tested with polyethylene glycol (PEG) 6000 seed osmopriming and drought stress.
- Significant variability is observed in various agronomic traits in response to different treatment levels.
- The combination of 20% SMC with −4 bar PEG was the most effective formulation for stimulating growth and enhancing the yield of Tadong upland rice.

## INTRODUCTION

Over the last few years, crop production across various ecosystems has been adversely affected by variations in environmental conditions, including abiotic stresses that significantly hinder growth and yield (Kopecká *et al*. 2023). Apart from food safety, the main issue with global agriculture is food security, where unstable and reduced crop yields are produced. This issue is a major concern considering the increase in global population and growing demand for better diets. Various techniques have been employed, ranging from genetic modification and seed treatments to field planting, to enhance yield; however, unpredictable and extreme weather events such as drought present significant challenges. Drought imposes additional stress on plants by reducing water availability, impairing nutrient uptake and inducing physiological and morphological changes such as leaf structure. This stress leads to significant disturbance to crops, either reducing yield or causing direct damage to the plant.

Research indicates that drought conditions can cause a reduction in leaf water potential and stomatal conductance, which in turn limits photosynthesis and overall plant vigour (Jarin *et al*. 2024). Additionally, drought stress has been shown to alter root architecture, promoting deeper root systems to enhance water uptake, although this adaptation may not fully compensate for the lack of moisture (Zhang *et al*. 2024). Moreover, drought can induce oxidative stress, resulting in increased production of reactive oxygen species (ROS), which can damage cellular components and impair metabolic functions (Salgotra & Chauhan 2023). However, the effectiveness of these physiological adaptations can vary significantly among different rice cultivars, highlighting the need for targeted breeding programmes to improve drought resilience (Zhu & Shen 2024).

The rice self-sufficiency level (SSL) in Sabah is currently estimated at approximately 22%, reflecting the proportion of locally produced rice relative to the total consumption demand of the state’s population (*Bernama* 2024). Upland rice, cultivated locally in Sabah, has potential due to its drought-adaptability properties and could be one of the best options for producing rice in environments with limited water (Sarma *et al*. 2023). It is typically grown with annual shifting cultivation practices, along with minimal use of agricultural inputs and a long maturation period of cultivation and is well adapted to the ecosystem’s unpredictable constraints (Sohrabi *et al*. 2012; Rahmat *et al*. 2014). The poor and ineffective crop management practices, including minimal input application, inadequate weed management, and abiotic and biotic stresses, have resulted in low yields of upland rice (Zenna *et al*. 2017). Due to its low yield, upland rice is primarily cultivated for domestic consumption within local communities, with surplus sold as a supplementary income source when demand is sufficient, and production exceeds local needs (Sinton *et al*. 2019). In the late 2000s, the average yield of upland rice remained low and unstable (Sohrabi *et al*. 2012; Ahmad *et al*. 2016), which calls for immediate action to address the shortage. The yield of upland rice can be enhanced by employing effective technologies for efficient production, implementing appropriate soil and plant management practices, and pursuing genetic improvement strategies (Saito *et al*. 2018).

Seed priming is a technique that involves soaking seeds in a solution followed by dehydration before sowing (Guan *et al*. 2009; Zhang *et al*. 2023). The technique can enhance early crop growth wherein the emergence and germination of some crop seeds are improved (Waqas *et al*. 2019; Zhang *et al*. 2023). This technology is recognised for promoting uniform and rapid seed emergence, enhancing germination rates and uniformity in heterogeneously maturing seed lots, thereby resulting in greater vigour, healthier plant establishment and improved crop yields (Devika *et al*. 2021; Adhikari *et al*. 2022). Uniform emergence and fast germination help farmers to “catch up” on the period lost to drought (Singh *et al*. 2014). Osmopriming is broadly used as a profitable method in which seed hydration is controlled to allow metabolic activities for pre-germination (Waqas *et al*. 2019).

Osmopriming with agents like polyethylene glycol (PEG), chitosan and calcium chloride (CaCl_2_) enhances seed vigour, root and shoot growth, and physiological performance under chilling stress by modulating antioxidant enzyme activities and increasing the accumulation of osmoprotectants like proline, soluble sugars and proteins. These biochemical changes reduce oxidative damage and lipid peroxidation, leading to better germination rates and seedling establishment under adverse conditions (Zhang *et al*. 2023). Similarly, osmopriming with PEG has been shown to improve seed germination and seedling physiological traits under water stress in *Coronilla varia* by enhancing water retention and protecting cellular integrity (Ma *et al*. 2024). Furthermore, proline osmopriming specifically improves root architecture, nitrogen content, and overall growth in rice seedlings, contributing to better nutrient uptake and biomass accumulation (Pereira *et al*. 2021). Collectively, these findings highlight osmopriming as a promising and practical approach to enhance seed performance and crop resilience against abiotic stresses in rice. Studying the effects of seed osmopriming and drought stress on upland rice is essential to understanding the mechanisms that enable the plant to resist drought. This study aimed to examine the effects of PEG 6000 seed osmopriming and drought stress on the growth, yield, physiological and biochemical parameters of Tadong upland rice.

## MATERIALS AND METHOD

### Experiment Site

The seed osmopriming and drought stress pot experiment was carried out in the insect-proof net house at the Faculty of Sustainable Agriculture (FSA), Universiti Malaysia Sabah. The experiment period was from February 2019 to November 2021.

### Tadong Upland Rice

The physical characteristics of the Tadong upland rice were described by Rou and Lum (2020). Generally, Tadong upland rice is an endemic upland rice variety, and the husk is brownish yellow, while the part without a husk is reddish black in colour. The rice is extra-long and medium in shape based on the scale by the International Rice Research Institute (IRRI) and has a moisture content of 12.11%.

### Experimental Design and Treatments

In this study, a 3 × 5 factorial experiment was arranged in a Completely Randomised Design (CRD). There was a total of 15 combination treatments with several drought stress levels using the soil moisture content (SMC) method of 30%, 20% and 10% SMC and osmopriming different PEG 6000 osmotic potential levels of 0, −2, −4, −6 and −8 bars. Each combination treatment comprised five replications, with a total of 75 pots used.

### Drought Stress Treatments

The drought stress levels were altered by utilising SMC. The treatments comprised soil drying slowly to specific moisture levels, followed by irrigation to maintain constant SMC. Treatments included 30% SMC (control) and drying to 20% and 10% SMC, with subsequent irrigation adjustments to maintain consistent moisture levels. The 30% SMC was selected as the control treatment because it represented the saturation point of the media mixture and was equivalent to 100% Field Capacity (FC). During the treatment period, soil moisture was monitored using a Field Scout Soil Sensor Reader to maintain intended levels. The treatment was applied at the panicle initiation stage and continued for 20 days (Davatgar *et al*. 2009). The irrigation regimes before and after the drought treatment remained constant, with no drought application to the rice plants.

### Seed Preparation and Transplanting

The Tadong upland rice seeds were obtained from Ranau, Sabah, Malaysia, and stored in a cool room with a temperature of 10°C to 15°C. The seed samples were taken randomly and primed with different osmotic potential levels of PEG 6000 for 48 h at 25°C. Floated seeds to the surface were removed. After the priming treatment, the treated upland rice seeds were cleaned and rinsed thoroughly using distilled water and dried for 24 h at 25°C. The seeds were germinated in a germination box at a room temperature of (24 ± 2°C) under dark conditions in the laboratory and left for 14 days. The 14-day-old seedlings of upland rice treated with different osmotic potential levels of PEG 6000 were transplanted into the planting pots, where each pot was transplanted with one rice seedling. The root of the seedling was moistened before transplanting. Each seedling was planted into the planting pot by making a hole with a depth of 10 cm, followed by pressing the soil gently once the seedling was planted to ensure that the seedling was properly implanted into the soil.

### Planting Media Preparation

The planting media used in this study was a mixture of Ultisol soil, compost and river sand with a ratio of 3:1:1, respectively, with a slight modification of the method from Sudarjat *et al*. (2018). Soil, sand, and compost were obtained from FSA and were air-dried under direct sunlight for a week, ground, and sieved individually before mixing. For the soil, Ultisol from a depth of 0 to 30 cm was obtained from FSA and included in the mixture.

### Fertiliser Application

A basal fertiliser comprising 60 kg of nitrogen (N) ha^−1^, 60 kg of phosphorus pentoxide (P_2_O_5_) ha^−1^, and 60 kg of potassium oxide (K_2_O) ha^−1^ by using NPK 15-15-15 fertiliser was applied to each pot one day prior to the transplanting of rice seedlings (Davatgar *et al*. 2009). For topdressing, 60 kg of nitrogen ha^−1^ was divided into two equal doses and application was performed at the mid-tillering and panicle initiation stages, respectively, using a urea straight fertiliser. The amount of fertiliser required for each pot was calculated according to the area of the soil in the pot. The application of fertiliser was performed at a depth of 3 cm from the soil surface and 5 cm from the upland rice plants, which were then covered by the soil to reduce the leaching of the fertiliser.

### Polyethylene Glycol 6000 Treatments

In this study, five different osmotic potential levels of PEG 6000 (0, −2, −4, −6 and −8 bars) were used for the priming of upland rice seeds (Lum *et al*. 2014; Rou & Lum 2020). After priming, the upland rice seeds were rinsed and dried at 25°C for 24 h. The amount of PEG 6000 required for each treatment was computed as follows:


$$\text{Osmotic potential}=-(1.18\times 10^{-2})\;\text{C}-(1.18\times 10^{-4})\;{\text{C}}^{2}+(2.67\times 10^{-4})\;\text{CT}+(8.39\times 10^{-7})\;{\text{C}}^{2}\text{T}$$

where OP = osmotic potential of PEG 6000, C = concentration of PEG 6000 in g/kg water and T = temperature.

### Parameters

The parameters of vegetative growth, yield, physiological characteristics and biochemical characteristics were assessed. Data on vegetative growth parameters were collected weekly during the study period, while rice panicles were harvested at physiological maturity to produce grains. For the vegetative growth components, the parameters studied included plant height, culm height, tiller number, leaf number per tiller, leaf length, leaf width, shoot dry weight, root dry weight, root-to-shoot ratio and root length. The measurement of yield components occurred when the paddy plants reached physiological maturity to produce grains. The yield component parameters included panicle number per plant, panicle length, percentage of productive tillers, grain number per panicle, percentage of filled grains per panicle, 1,000-grain weight and yield per plant. Physiological characteristics data collected for this study were leaf area index, relative water content, and relative chlorophyll content. Biochemical characteristics data collected for this study were free proline content (FPC) in leaf and root, and membrane stability index (MSI). Parameters on rice of physiological and biochemical characteristics and yield were collected after harvesting the rice grains.

### Statistical Analysis

The collected data underwent Factorial Analysis of Variance (ANOVA) using Statistical Analysis Software (SAS) Version 9.4 (SAS Institute Incorporation 2002). The Least Significant Difference (LSD) test at the 0.05 significance level was used to compare means when necessary.

## RESULTS

### Vegetative Growth of Tadong Upland Rice

The results indicate that SMC significantly influenced plant height (PH) and leaf length (LL) of Tadong upland rice (*p* < 0.01) (Table 1). Specifically, PH decreased from 153.48 cm at 30% SMC to 149.12 cm at 10% SMC, showing a clear trend of reduced cell elongation and growth under drought stress. Similarly, LL increased in some osmopriming treatments, with a notable rise at −2 bar PEG (69.81 cm) compared to unprimed plants (64.72 cm), highlighting the beneficial effects of PEG priming on seedling vigour under drought. Culm height (CH), tiller number (TN), leaf number per tiller (LNT), leaf width (LW), shoot dry weight (SDW), root dry weight (RDW), root-shoot ratio (RSR) and root length (RL) did not consistently show significant differences with reduced soil moisture or PEG treatments, indicating that these traits might be less sensitive or more variable in response. However, there are significant interactions between SMC and PEG treatments for CH, TN, LW, LL and RL (*p* < 0.05). For PEG treatment effects, PH and LH were significantly affected (*p* < 0.01), emphasising the positive effect of seed osmopriming on vegetative growth, possibly by accelerating metabolism and imbibition speed. Root growth parameters mostly showed non-significant differences among treatments but suggested a slight improvement with osmopriming.

### Yield Component of Tadong Upland Rice

Yield components were markedly influenced by SMC and PEG priming. The number of grains per panicle (GNP) decreased significantly from 136.80 at 30% SMC to 133.48 at 10% SMC (*p* < 0.05) (Table 2). More notably, the percentage of filled grains per panicle (PFGP) decreased sharply under drought, from 69.24% at 30% SMC to 56.57% at 10% SMC (*p* < 0.01), indicating impaired reproductive success under water stress. Panicle number per plant (PNP), panicle length (PL) and percentage of productive tillers (PPT) did not show significant decreases with drought, suggesting relative stability in these yield components. PEG priming significantly affected PNP (*p* < 0.05), PFGP (*p* < 0.01), and to a lesser extent of GNP, demonstrating that seed priming can mitigate yield losses by improving grain filling and fertility. Yield per plant (YP) also varied significantly, with the highest yield under the −4 bar PEG osmopriming (741.35 g/m^2^), reflecting better drought tolerance through priming. Despite being significant for either drought, PEG or both, a significant interaction between drought stress and PEG treatment was observed for PNP (*p* < 0.05), PFGP (*p* < 0.05), and YP (*p* < 0.01), suggesting that the influence of drought stress on these key yield components is contingent upon the level of PEG osmopriming applied.

### Physiological Characteristics of Tadong Upland Rice

Relative water content (RWC) was not significantly affected by SMC but increased with higher PEG priming pressure, peaking at 90.96% in plants primed with −8 bar PEG (*p* < 0.01) (Table 3). This indicates improved water retention and osmotic adjustment due to priming. Leaf area index (LAI) was significantly reduced by drought stress (*p* < 0.01), with values declining from 0.44 at 30% SMC to 0.38 at 10% SMC. PEG treatments also significantly influenced LAI (*p* < 0.01), suggesting an osmopriming role in maintaining leaf expansion and photosynthetic capacity under stress. Relative chlorophyll content (RCC) decreased significantly at the lowest SMC (20.29 at 10% SMC vs. ~23 at higher), suggesting pigment degradation and impaired photosynthetic machinery due to drought stress. However, a significant interaction between drought stress and PEG treatment (*p* < 0.05) was observed for LAI and RCC, demonstrating that the drought-induced changes in these physiological parameters are influenced by the degree of PEG osmopriming applied.

### Biochemical Responses of Tadong Upland Rice

Root free proline content (RFPC) also showed significant decreases under drought and priming in some treatments (*p* < 0.01) (Table 4). Leaf free proline content (LFPC) increased significantly under drought stress, from 15.22 mmol/g FW at 30% SMC to about 24.62 mmol/g FW at 20% SMC (*p* < 0.01). PEG priming further elevated proline levels, especially at −2 bar PEG (29.39 mmol/g FW), demonstrating enhanced osmolyte accumulation critical for drought resilience. However, a significant interaction between drought stress and PEG treatment was observed (*p* < 0.01). Similarly, a significant interaction between drought stress and PEG was detected for the membrane stability index (MSI). MSI, an indicator of cell membrane integrity under stress, was significantly affected by both SMC and PEG treatments (*p* < 0.05). Plants primed with −2 bar PEG exhibited the highest MSI value (19.67%), indicating improved membrane protection and antioxidant defense activated by priming.

## DISCUSSION

### Effect of Drought Stress and Seed Osmopriming on Vegetative Growth of Tadong Upland Rice

Drought stress profoundly affects rice development throughout its life cycle, inducing physiological and morphological changes that compromise growth and yield potential. In this study, a significant reduction in PH was observed under decreasing SMC, from 153.48 cm at 30% SMC to 149.12 cm at 10% SMC (*p* < 0.01, Table 1). This reduction aligns with the known drought-induced restriction of cell elongation due to lowered turgor pressure, which subsequently decreases internode elongation and limits node production (Patel *et al*. 2010; Kondhia *et al*. 2015).

Significant interactions between drought stress and PEG levels were detected for CH, TN, LW, LL, and RL (*p* < 0.05 to *p* < 0.01), demonstrating that the impact of drought on these traits is dependent on the degree of osmopriming. For example, although PH generally decreased with decreasing SMC—from 153.48 cm at 30% SMC to 149.12 cm at 10% SMC—the primed plants, especially those treated with intermediate PEG concentrations (−2 and −4 bar), maintained or enhanced growth traits such as leaf length (up to 69.81 cm at −2 bar PEG) and leaf width, compared to non-primed plants. This suggests that PEG priming alleviated drought-induced growth limitations, likely by stimulating early seed metabolism and triggering adaptive stress memory processes that improve cell expansion and elongation under water deficit (Chen & Arora, 2013; Ahmed *et al*. 2021).

Reduction in CH and plant biomass under water deficit is attributed to diminished photosynthetic efficiency and inhibited cell division caused by limited water uptake (Rahim *et al*. 2012). The observed trends in TN and LNT, though statistically non-significant, suggest drought places physiological constraints on shoot branching and leaf production that may become pronounced over longer stress durations or in different genotypes (Cerqueira *et al*. 2013).

Root traits, critical for drought adaptation, showed interesting patterns. While root dry weight slightly decreased with lower moisture, the root-shoot ratio remained statistically stable (Table 1), indicating a possible balance in biomass partitioning to maintain water uptake efficiency despite overall growth reduction. This finding concurs with reports that root:shoot allocation may be genotype- and environment-specific, with some studies noting increased root-shoot ratios as a survival strategy under drought (Akhtar & Nazir 2013; Brunner *et al*. 2015; Kul *et al*. 2020).

Seed osmopriming with PEG 6000 clearly enhanced vegetative growth traits. Significant increases in leaf length and maintenance of plant height under priming treatments indicate that priming accelerates seed metabolism and imbibition, offering seedlings a more vigorous start and enabling better establishment under water-limited conditions (Patanè *et al*. 2009; Ahmed *et al*. 2021).

### Effect of Drought Stress and Seed Osmopriming on Yield Component of Tadong Upland Rice

The yield components of Tadong upland rice were significantly influenced by drought stress as well as by seed osmopriming with PEG, with notable interaction effects between these two factors. While SMC alone affected certain yield traits, including GNP and PFGP, the interaction between drought stress and PEG priming revealed that the magnitude and direction of drought effects on yield components depended on the level of osmopriming applied. Effective grain production is sensitive to water availability during reproductive development. Yield component analysis revealed significant declines in grain number per panicle and percentage of filled grains under drought. Specifically, grain number decreased from 136.80 to 133.48, and filled grain percentage dropped substantially from 69.24% to 56.57% between 30% and 10% SMC (Table 2). These decreases suggest drought-mediated disruption to floral organ development and spikelet fertility, which restrict carbohydrate allocation and impair grain filling—a phenomenon well-documented in rice drought physiology (Behera *et al*. 2017; Zhang *et al*. 2018; Mukamuhirwa *et al*. 2019).

Panicle length and productive tiller percentage were relatively stable across treatments, indicating that these attributes may be less drought-sensitive or compensatory mechanisms may preserve them. These stable traits could provide critical yield support when other components decline under drought (Shrestha *et al*. 2021). Seed osmopriming alleviated yield reductions by enhancing grain filling and fertility, as reflected in the highest yield per plant observed at −4 bar PEG priming (741.35 g/m²), exceeding unprimed controls even under stress. Priming likely activates enhanced enzymatic activity and carbohydrate mobilisation, supporting fertilisation and grain development during water stress (Cao *et al*. 2018; Bhadouriya *et al*. 2021; Liu *et al*. 2021).

The 1,000-grain weight did not significantly decline but showed a downward trend consistent with the genetically determined maximum grain size limits, indicating that drought mainly affected grain filling rather than grain size potential (Dou *et al*. 2016; Sehgal *et al*. 2018). The detrimental impact of drought on photosynthetic machinery during flowering further exacerbates yield losses by limiting assimilates for grain growth (Fahad *et al*. 2017; Bahuguna *et al*. 2018). The 1,000-grain weight demonstrated a significant decline with increasing drought severity but was less influenced by PEG treatment or the interaction, underscoring the genetic control and physical constraints on grain size. Nonetheless, the combined positive effects of PEG priming on panicle number and grain filling collectively contributed to higher overall yield per plant despite drought stress. These findings strongly support the role of PEG osmopriming as a pragmatic agronomic strategy for improving drought tolerance in upland rice by enhancing the yield stability.

### Effect of Drought Stress and Seed Osmopriming on Physiological Characteristics of Tadong Upland Rice

Leaf area index exhibited a significant reduction with decreasing SMC, declining from 0.44 at 30% SMC to 0.38 at 10% SMC (*p* < 0.01), reflecting the detrimental effect of water deficit on leaf expansion and canopy development. PEG osmopriming further modulated this response, with primed seeds showing a tendency to maintain higher LAI under drought conditions, suggesting that osmopriming mitigates drought-induced restrictions on leaf growth through enhanced water uptake and metabolic activation (Ahmed *et al*. 2021). The significant interaction between SMC and PEG treatment for LAI (*p* < 0.05) indicates that the degree of PEG priming influences how drought stress impacts leaf area development. Lower leaf area constrains carbon fixation, exacerbating growth and yield deficits (Verma *et al*. 2019; Hernandez *et al*. 2021).

Relative water content of leaves is a pivotal indicator of cellular hydration and drought tolerance. While RWC did not show significant SMC effects, PEG priming notably increased leaf RWC, with −8 bar PEG treatment achieving the highest values (90.96%), implying improved tissue hydration and osmotic adjustment that support metabolic activity during drought (Gupta & Guhey 2011; Mishra *et al*. 2019).

Relative chlorophyll content was also significantly affected by the interaction between drought and PEG priming (*p* < 0.05). Drought stress alone reduced chlorophyll content, likely due to impaired pigment biosynthesis and increased degradation under water deficit (Pandey & Shukla 2015). However, PEG-primed plants showed relatively higher RCC values under drought, indicating better preservation of photosynthetic pigments and enhanced photosynthetic efficiency. This protective effect may be attributed to PEG-induced stress memory and antioxidant system activation, which stabilise chloroplast function and reduce oxidative damage (Günay *et al*. 2022). Reduced chlorophyll content at low moisture underscores drought-induced pigment degradation and impaired photochemical efficiency, critical for limiting photosynthetic carbon assimilation (Maisura *et al*. 2014).

### Effect of Drought Stress and Seed Osmopriming on Biochemical Responses of Tadong Upland Rice

Proline accumulation, particularly in leaves, is a well-recognised adaptive response to osmotic stress, acting as an osmoprotectant, reactive oxygen species (ROS) scavenger, and stabiliser of cellular structures (Al-Ashkar *et al*. 2016). In this study, drought stress significantly elevated LFPC, increasing from 15.22 mmol/g FW at 30% SMC to 24.62 mmol/g FW at 20% SMC and 23.84 mmol/g FW at 10% SMC. PEG priming further enhanced proline accumulation, with the highest LFPC observed at −2 bar PEG treatment (29.39 mmol/g FW), suggesting that osmopriming primes the stress memory mechanisms, enabling rice plants to pre-emptively synthesise higher proline levels and better cope with subsequent drought stress (Liu *et al*. 2021; Yang *et al*. 2021; Mahmud *et al*. 2023). The interaction between SMC and PEG was significant for LFPC, indicating that the effect of drought on proline biosynthesis is strongly influenced by the priming level. Such an interaction underscores the enhanced biochemical resilience imparted by PEG priming, which facilitates better osmotic adjustment and cellular protection under water deficit.

The MSI was significantly modulated by the interaction between drought and PEG osmopriming. Drought stress alone reduced MSI (from 17.80% at 30% SMC to 14.58% at 10% SMC), indicating increased membrane damage under water deficit. However, PEG priming markedly improved MSI values, with primed plants showing higher membrane stability at comparable SMC levels, especially at −2 and −6 bar PEG treatments. This protective effect is attributed to enhanced antioxidant defenses and ROS scavenging induced by PEG priming, reducing lipid peroxidation and membrane injury during drought (Mishra *et al*. 2019; Günay *et al*. 2022).

Root-free proline content was highest in roots under well-watered conditions (30% SMC) and decreased progressively with increasing drought severity, indicating that proline accumulation in roots may not follow a linear increase under drought stress and could be influenced by complex physiological or cultivar-specific responses. PEG priming also significantly altered RFPC, with the greatest proline accumulation observed at −6 bar PEG, suggesting that osmopriming enhances the root’s capacity to produce proline and thus boosts its drought tolerance potential. This priming effect likely stems from the activation of stress-responsive metabolic pathways before exposure to actual drought, enabling primed seedlings to mount a more effective biochemical defense upon stress onset (Mahmud *et al*. 2023).

## CONCLUSION

The Tadong upland rice demonstrates potential for successful cultivation, particularly in drought-prone areas. The integration of 20% SMC combined with −4 bar PEG proved to be the most effective formulation for promoting growth and enhancing yield. This specific combination resulted in a noteworthy increase of 55.68%, highlighting its potential for maximising agricultural productivity even under drought conditions. Research indicates that various agronomic traits, including plant height, number of grains per panicle, weight of 1,000 grains and root-free proline content, show significant variability in response to different treatment levels. This suggests that these traits are sensitive to changes and can be effectively measured to assess drought conditions’ impact on paddy performance. Furthermore, when Tadong upland rice is osmo-primed using PEG 6000, notable improvements are observed in several important parameters. Specifically, this treatment leads to significant increases in plant height, relative water content, number of grains per panicle, and root-free proline content. These findings indicate that osmo-priming not only enhances Tadong rice’s resilience to drought but also improves its overall growth performance and yield potential.

## Figures and Tables

**TABLE 1:** Growth responses of Tadong upland rice in different soil moisture contents and polyethylene glycol 6000 at 124 DAS.

Treatment	PH (cm)	CH (cm)	TN	LNT	LL (cm)	LW (cm)	SDW (g)	RDW (g)	RSR	RL (cm)
SMC (%)										
30	153.48^a^ ± 5.82	141.20^a^ ± 8.34	17.00^a^ ± 2.22	3.64^a^ ± 0.57	64.72^b^ ± 5.41	2.07^a^ ± 0.128	121.43^a^ ± 15.26	17.43^a^ ± 3.81	0.14^a^ ± 0.02	38.74^a^ ± 4.90
20	150.42^b^ ± 4.64	142.10^a^ ± 7.73	16.52^a^ ± 3.39	3.48^a^ ± 0.77	64.84^b^ ± 5.26	2.12^a^ ± 0.132	116.32^ab^ ± 14.76	14.76^b^ ± 3.80	0.13^b^ ± 0.02	35.23^b^ ± 5.75
10	149.12^b^ ± 6.19	139.52^a^ ± 6.37	17.16^a^ ± 3.00	3.32^a^ ± 0.63	69.22^a^ ± 7.89	2.07^a^ ± 0.130	111.88^b^ ± 12.34	16.03^ab^ ± 5.19	0.14^ab^ ± 0.04	39.02^a^ ± 5.01
PEG (bar)										
0	151.53^a^ ± 7.82	139.14^a^ ± 8.63	16.73^a^ ± 2.79	3.60^a^ ± 0.83	64.87^bc^ ± 7.19	2.04^a^ ± 0.14	117.59^a^ ± 15.38	16.02^a^ ± 4.99	0.13^a^ ± 0.03	39.19^a^ ± 4.88
−2	153.60^a^ ± 4.19	142.33^a^ ± 6.39	16.53^a^ ± 3.09	3.47^a^ ± 0.52	69.81^a^± 7.37	2.12^a^ ± 0.10	114.63^a^ ± 10.74	16.03^a^ ± 2.93	0.14^a^ ± 0.02	36.36^a^ ± 4.81
−4	152.20^a^ ± 6.44	140.15^a^ ± 7.84	18.07^a^ ± 2.12	3.47^a^ ± 0.52	66.68^ab^ ± 4.91	2.06^a^ ± 0.14	120.04^a^ ± 15.26	17.17^a^ ± 5.70	0.14^a^ ± 0.04	36.57^a^ ± 6.87
−6	150.83^a^ ± 4.22	143.13^a^ ± 7.97	15.40^a^ ± 2.95	3.60^a^ ± 0.51	62.91^c^ ± 6.78	2.12^a^ ± 0.12	111.30^a^ ± 17.51	14.96^a^ ± 3.56	0.13^a^ ± 0.03	38.00^a^ ± 4.76
−8	146.87^b^ ± 3.64	139.95^a^ ± 6.71	17.73^a^ ± 2.94	3.27^a^ ± 0.88	67.03^ab^ ± 4.84	2.09^a^ ± 0.15	119.16^a^ ± 12.98	16.20^a^ ± 4.57	0.13^a^ ± 0.03	38.20^a^ ± 5.82
SMC	**	NS	NS	NS	**	NS	NS	NS	NS	**
PEG	**	NS	NS	NS	**	NS	NS	NS	NS	NS
SMC × PEG	NS	*	*	NS	**	*	NS	NS	NS	**
LSD 5% SMC	2.82	4.02	1.48	0.39	2.73	0.07	8.00	2.43	0.016	2.52
LSD 5% PEG	3.64	5.19	1.91	0.51	3.52	0.09	10.32	3.14	0.021	3.26

*Notes:* Data are means ± standard deviation. Values in each column followed by different letters indicate significant differences according to the LSD test (probability level of 5%). SMC = Soil moisture content; PEG = Polyethylene glycol; PH = Plant height; CH = Culm height; TN = Tiller number; LNT = Leaf number per tiller; LL = Leaf length; LW = Leaf width; SDW = Shoot dry weight; RDW = Root dry weight; RSR = Root-shoot ratio; RL = Root length.

* Significant at 5% level of probability;

** Significant at 1% level of probability; NS = not significant

**TABLE 2:** Yield responses of Tadong upland rice in different soil moisture contents and polyethylene glycol 6000.

Treatment	PNP	PL (cm)	PPT (%)	GNP	PFGP (%)	TGW (g)	YP (g/m^2^)
SMC (%)							
30	16.60^a^ ± 2.24	30.46^a^ ± 0.99	97.72^a^ ± 4.33	136.80^ab^ ± 13.03	69.24^a^ ± 9.11	40.11^a^ ± 2.12	747.50^a^ ± 100.27
20	15.96^a^ ± 3.21	30.74^a^ ± 1.86	96.85^a^ ± 5.75	144.28^a^ ± 19.90	64.47^b^ ± 8.38	38.57^b^ ± 2.80	670.77^b^ ± 95.55
10	16.96^a^ ± 2.96	30.11^a^ ± 1.71	98.84^a^ ± 2.43	133.48^b^ ± 15.78	56.57^c^ ± 11.20	36.99^c^ ± 2.48	557.74^c^ ± 106.75
PEG (bar)							
0	16.53^ab^ ± 2.85	30.11^a^ ± 2.01	98.80^a^ ± 3.34	135.47^b^ ± 10.44	66.89^a^ ± 6.00	38.51^a^ ± 4.00	689.32^ab^ ± 118.71
−2	16.00^ab^ ± 2.75	30.46^a^ ± 1.49	97.19^a^ ± 7.03	135.07^b^ ± 18.62	57.68^b^ ± 12.69	38.24^a^ ± 2.85	568.45^d^ ± 140.43
−4	17.73^a^ ± 1.94	30.17^a^ ± 1.20	98.28^a^ ± 2.53	134.27^b^ ± 9.74	69.60^a^ ± 6.95	37.69^a^ ± 2.31	741.35^a^ ± 103.06
−6	14.87^b^ ± 2.67	31.20^a^ ± 1.70	96.84^a^ ± 4.84	150.73^a^ ± 22.73	65.94^a^ ± 12.04	39.01^a^ ± 2.34	672.64^bc^ ± 110.74
−8	17.40^a^ ± 3.16	30.24^a^ ± 1.25	97.91^a^ ± 3.09	135.40^b^ ± 15.08	57.01^b^ ± 9.70	39.33^a^ ± 1.91	621.59^cd^ ± 97.66
SMC	NS	NS	NS	*	**	**	**
PEG	*	NS	NS	*	**	NS	**
SMC × PEG	*	NS	NS	NS	*	NS	**
LSD 5% SMC	1.44	0.89	2.52	8.69	4.29	1.41	42.24
LSD 5% PEG	1.86	1.15	3.25	11.22	5.53	1.82	54.53

*Notes:* Data are means ± standard deviation. Values in each column followed by different letters indicate significant differences according to the LSD test (probability level of 5%). SMC = Soil moisture content; PEG = Polyethylene glycol; PNP = Panicle number per plant; PL = Panicle length; PPT = Percentage of productive tillers; GNP = Grain number per panicle; PFGP = Percentage of filled grains per panicle; TGW = 1000-grain weight; YP = Yield per plant.

* Significant at 5% level of probability;

** Significant at 1% level of probability; NS = not significant.

**TABLE 3:** Physiological characteristics of Tadong upland rice in different soil moisture contents and polyethylene glycol 6000.

Treatment	LAI	RWC (%)	RCC (SPAD unit)
SMC (%)			
30	0.44^a^ ± 0.05	88.27^a^ ± 2.52	22.82^ab^ ± 8.92
20	0.44^a^ ± 0.03	88.29^a^ ± 2.51	26.78^a^ ± 9.54
10	0.38^b^ ± 0.05	88.43^a^ ± 3.47	20.29^b^ ± 10.79
PEG (bar)			
0	0.44^a^ ± 0.06	88.64^b^ ± 2.63	23.67^a^ ± 11.43
−2	0.45^a^ ± 0.03	87.53^b^ ± 3.16	23.47^a^ ± 11.28
−4	0.43^ab^ ± 0.05	87.44^b^ ± 2.27	23.12^a^ ± 9.42
−6	0.41^b^ ± 0.03	87.07^b^ ± 1.82	21.60^a^ ± 7.37
−8	0.38^c^ ± 0.05	90.96^a^ ± 2.51	24.61^a^ ± 11.14
SMC	**	NS	*
PEG	**	**	NS
SMC × PEG	*	NS	*
LSD 5% SMC	0.019	1.51	5.17
LSD 5% PEG	0.025	1.95	6.67

*Notes*: Data are means ± standard deviation. Values in each column followed by different letters indicate significant differences according to the LSD test (probability level of 5%). SMC = Soil moisture content; PEG = Polyethylene glycol; LAI = Leaf area index; RWC = Relative water content, RCC = Relative chlorophyll content;

* Significant at 5% level of probability;

** Significant at 1% level of probability; NS = not significant.

**TABLE 4:** Biochemical responses of Tadong upland rice in different soil moisture contents and polyethylene glycol 6000.

Treatment	LFPC (mmole/g FW)	RFPC (mmole/g FW)	MSI (%)
SMC (%)			
30	15.22^b^ ± 4.73	5.76^a^ ± 0.83	17.80^a^ ± 5.72
20	24.62^a^ ± 8.81	5.31^a^ ± 1.68	16.97^a^ ± 4.87
10	23.84^a^ ± 10.74	4.18^b^ ± 1.34	14.58^b^ ± 3.94
PEG (bar)			
0	25.47^b^ ± 7.59	5.21^b^ ± 1.01	12.46^b^ ± 4.33
−2	29.39^a^ ±9.09	4.98^b^ ± 1.17	19.67^a^ ± 3.97
−4	14.17^e^ ± 3.18	5.20^b^ ± 1.73	14.55^b^ ± 4.85
−6	20.94^c^ ± 11.79	6.12^a^ ± 1.06	18.05^a^ ± 4.95
−8	16.17^d^ ± 2.37	3.92^c^ ± 1.52	17.53^a^ ± 3.85
SMC	**	**	*
PEG	**	**	**
SMC × PEG	**	NS	*
LSD 5% SMC	1.34	0.62	2.24
LSD 5% PEG	1.73	0.80	2.89

*Notes:* Data are means ± standard deviation. Values in each column followed by different letters indicate significant differences according to the LSD test (probability level of 5%). SMC = Soil moisture content; PEG = Polyethylene glycol; LFPC = Leaf free proline content; RFPC = Root free proline content; MSI = Membrane stability index.

* Significant at 5% level of probability;

** Significant at 1% level of probability; NS = not significant.
